# Microbiota changes associated with ADNP deficiencies: rapid indicators for NAP (CP201) treatment of the ADNP syndrome and beyond

**DOI:** 10.1007/s00702-020-02155-5

**Published:** 2020-02-18

**Authors:** Oxana Kapitansky, Eliezer Giladi, Iman Jaljuli, Stefan Bereswill, Markus M. Heimesaat, Illana Gozes

**Affiliations:** 1 The First Lily and Avraham Gildor Chair for the Investigation of Growth Factors, Dr. Diana and Zelman Elton (Elbaum) Laboratory for Molecular Neuroendocrinology, Department of Human Molecular Genetics and Biochemistry, Sackler Faculty of Medicine, Sagol School of Neuroscience and Adams Super Center for Brain Studies, Tel Aviv University, 69978 Tel Aviv, Israel; 2 Department of Statistics and Operations Research, School of Mathematical Sciences, Raymond and Beverly Sackler Faculty of Exact Sciences, Tel-Aviv University, 69978 Tel Aviv, Israel; 3 Gastrointestinal Microbiology Research Group, Institute for Microbiology, Infectious Diseases and Immunology, Charité-University Medicine Berlin, Hindenburgdamm 30, 12203 Berlin, Germany

**Keywords:** Activity-dependent neuroprotective protein (ADNP), Microbiota, NAP (CP201), Behavior, Autism

## Abstract

Activity-dependent neuroprotective protein (ADNP) and its protein snippet NAP (drug candidate CP201) regulate synapse formation and cognitive as well as behavioral functions, in part, through microtubule interaction. Given potential interactions between the microbiome and brain function, we now investigated the potential effects of the ADNP-deficient genotype, mimicking the ADNP syndrome on microbiota composition in the *Adnp*^+/–^ mouse model. We have discovered a surprising robust sexually dichotomized *Adnp* genotype effect and correction by NAP (CP201) as follows. Most of the commensal bacterial microbiota tested were affected by the Adnp genotype and corrected by NAP treatment in a male sex-dependent manner. The following list includes all the bacterial groups tested—labeled in bold are male *Adnp*—genotype increased and corrected (decreased) by NAP. (1) **Eubacteriaceae (EubV3)**, (2) Enterobacteriaceae (Entero), (3) *Enterococcus* genus (gEncocc), **(4) *Lactobacillus* group (Lacto), (5) *Bifidobacterium* genus (BIF)**, (6) *Bacteroides/Prevotella* species (Bac), (7) *Clostridium coccoides* group (Coer), (8) *Clostridium leptum* group (Cluster IV, sgClep), and **(9) *Mouse intestinal Bacteroides* (MIB).** No similarities were found between males and females regarding sex- and genotype-dependent microbiota distributions. Furthermore, a female *Adnp*^+/–^ genotype associated decrease (contrasting male increase) was observed in the *Lactobacillus* group (Lacto). Significant correlations were discovered between specific bacterial group loads and open-field behavior as well as social recognition behaviors. In summary, we discovered ADNP deficiency associated changes in commensal gut microbiota compositions, a sex-dependent biomarker for the ADNP syndrome and beyond. Strikingly, we discovered rapidly detected NAP (CP201) treatment-dependent biomarkers within the gut microbiota.

**Electronic supplementary material:**

The online version of this article (10.1007/s00702-020-02155-5) contains supplementary material, which is available to authorized users.

## Introduction

Autism spectrum disorder (ASD) constitutes a complex neurodevelopmental disorder that encompasses a wide array of symptoms ranging from sensory sensitivity, social anxiety, and communication difficulties, to repetitive behaviors. In addition, children diagnosed with ASD display various medical comorbidities of high prevalence (Banaschewski et al. 2011; Geier et al. 2012; Ozsivadjian et al. 2013). For example, previous research strongly implicates a role for metabolites derived from the gut microbiota as modulators of autistic behavioral phenotypes, influencing the host metabolome and shaping disease outcomes, at the individual level (Mussap et al. 2016).

The prevalence of ASDs has been increasing over the past 2 decades at an alarming rate. According to a recent review of world medical records, 1 in 68 children aged 8 years were identified as having ASD (Christensen et al. 2016). Despite the obvious prevalence, little is known about the underlying molecular mechanisms of immunopathogenesis. Neuroimaging and *postmortem* studies have provided evidence for disruptions in functional and structural connectivity in the brains of affected individuals (Vissers et al. 2012). Autism presents a great challenge to science; currently, large genes that are crucial for brain development serve as key candidates for research on autism pathophysiology (Larsen et al. 2016). In this respect, while searching for genes that shape our brains, we discovered the activity-dependent neuroprotective protein (ADNP), a highly conserved, vertebrate-specific protein, essential for brain formation and function. ADNP is a ~ 124 kDa protein (coded by the human *ADNP* gene on chromosome 20q12-13.2) (Bassan et al. 1999; Gozes et al. 2015b; Zamostiano et al. 2001) that is necessary for brain development, brain plasticity, and cognitive and social functioning, all of which may be impaired in ASD (Amram et al. 2016; Malishkevich et al. 2015; Pinhasov et al. 2003; Vulih-Shultzman et al. 2007). Recently, heterozygous dominant, disease inflicting de novo mutations in *ADNP* have been identified in cohorts of intellectually disabled (ID) children suffering from syndromic ASD and calculated to affect 0.17% of the autistic children—defining the ADNP syndrome (Gozes et al. 2015a, 2017a, b; Helsmoortel et al. 2014; Levine et al. 2019; Mollinedo et al. 2019; O'Roak et al. 2012) (OMIM 611386 and Orphanet—https://www.orpha.net/consor/cgi- bin/OC_Exp.php?Lng = EN&Expert = 404448). Importantly, *ADNP* is one of a group of de novo mutated genes including *CHD8*, *TBR1, SYNGAP1, and SHANK3* that lead to autism in a substantial proportion of cases (Deciphering Developmental Disorders 2017; Larsen et al. 2016), with some similar fundamental mechanisms affecting synaptic function and phenotypic characteristics.

Upon discovery of *ADNP*, we also identified an eight amino acid peptide fragment, namely NAP (NAPSVIPQ) (Bassan et al. 1999). This peptide includes a microtubule (MT) end-binding protein motif (SxIP) (Oz et al. 2014), fortifying the interaction of ADNP with MTs, enhancing MT dynamics and autophagy, protecting against electrical blockade, and showing cognitive protection in preclinical and clinical studies (Bassan et al. 1999; Gozes et al. 2017a).

Our recent results revealed that *Adnp*^+/–^ mice have developmental delays, impaired vocalizations, and motor dysfunction along with memory and social impairments, mimicking the ADNP syndrome in children (Hacohen-Kleiman et al. 2018). Exogenous administration of NAP was shown to at least partially reverse behavioral and developmental defects (Hacohen-Kleiman et al. 2018). In a different experimental model (i.e., gut inflammation), anti-inflammatory NAP effects were observed in human microbiota-harboring mice suffering from subacute ileitis. In this mouse model, we showed that NAP treatment potentially increased probiotic commensal bifidobacterial loads in the intestinal tract (Escher et al. 2018), suggesting a correlation between the autistic/intellectually disabled brain and the gut microbiota. In this respect, about 80% of the ADNP children suffer from problems within their digestive tract (Van Dijck et al. 2019).

Mechanistically, gamma-aminobutyric acid (GABA), the primary inhibitory neurotransmitter in the adult brain, has been implicated in autism etiology. A number of studies have consistently found reductions in specific subtypes of the GABA receptors in the cortex and hippocampus in a consistent deficit in autism (Blatt et al. 2001; Fatemi et al. 2009; Guptill et al. 2007; Ma et al. 2005). Importantly, tubulin β3, the most dynamic tubulin (Xu et al. 2017), associated with NAP/ADNP (Divinski et al. 2006) interacts with the GABAA receptor-associated protein, which is known to be involved in GABAA receptor trafficking (Xu et al. 2017). Pertinent to the microbiome involvement hypothesis, *Lactobacillus reuteri*, a commensal bacterial species with decreased relative abundance in the autism-lined Shank3 knockout mice microbiome, positively correlated with the expression of GABA receptor subunits in the brain. Treatment of Shank3 knockout mice with *L. reuteri* induced an attenuation of unsocial behavior (in male Shank3 knockout mice), and a decrease in repetitive behaviors in both sexes. Furthermore, *L. reuteri* treatment affected GABA receptor expression in the brain (Tabouy et al. 2018).

The excitatory, glutamatergic synapses are mainly located on the dendrites of principal neurons as small protrusions called dendritic spines (McKinney 2010). Dendritic spine dysgenesis is often reported in individuals and animal models of ASD (including atypical spine numbers and morphologies such as more immature in shape and less large-shaped spines) (Bernardinelli et al. 2014; Fiala et al. 2002; Phillips and Pozzo-Miller 2015). Importantly, we most recently showed that ADNP/NAP regulate the glutamatergic synapse and ameliorate dendritic spine dysregulation (Hacohen-Kleiman et al. 2018; Sragovich et al. 2019).

In the present study, we asked whether ADNP mutations at the individual level correlate with distinct differences in gut microbiota composition and whether exogenous NAP application ameliorates these ADNP related deficiencies. In the context of other potentially related models of autism, we hypothesize an ADNP dependent effect on the microbiota composition and potential brain-gut cross-talk related effects, which in a future translational perspective might add to a patient stratification biomarker (Tabouy et al. 2018).

## Materials and methods

### Animals

All procedures involving animals in the *Adnp*-mutated mouse model were approved by the Animal Care and Use Committee of Tel Aviv University and the Israeli Ministry of Health. *Adnp* heterozygous mice (*Adnp*^+/–^) on an ICR background (an outbred mouse line), a model for the ADNP syndrome (Hacohen-Kleiman et al. 2018), were housed in a 12-h light/12-h dark cycle facility with free access to rodent chow and water. Genotyping was performed by Transnetyx (Memphis, Tennessee). After genotyping, the mice were housed in separate cages based on sex, genotype, and treatment (up to five mice per cage), for 2 weeks prior to beginning of NAP treatment.

### Peptide synthesis and NAP treatment

NAP peptide was custom made as before (Hacohen-Kleiman et al. 2018). Prior to behavioral tests, intranasal treatment was administered daily to 1-month-old male and female mice (0.5 μg/5 μl/mouse/dose). For intranasal administration, the peptide was dissolved in a vehicle solution, termed DD, in which each milliliter included 7.5 mg of NaCl, 1.7 mg of citric acid monohydrate, 3 mg of disodium phosphate dihydrate, and 0.2 mg of benzalkonium chloride solution (50%). Each mouse was handheld in a semi-supine position with nostrils facing the investigator. A pipette tip was used to administer 5 μl/two nostrils. The mouse was handheld until the solution was entirely absorbed (~ 10 s). Nasal NAP application was performed daily, once a day, for 45 days (5 days a week). After 45 days of treatment, in days of scheduled behavioral tests, NAP was applied 2 h before the test.

Our original findings assessing the pharmacokinetics of NAP appearance and residence in the cerebrospinal fluid following intranasal administration compared to intravenous injection showed by specific pe Sciex api 4000 MS/MS system equipped with an Agilent 1100 HPLC that NAP (also known as davunetide or CP201) penetrates the brain mostly systemically through nasal blood vessel access, rather than by direct nose-brain transport (Morimoto et al. 2009). We have further proven this also by monitoring fluorescently labeled NAP by the Maestro machine (Cri MaestroTM in vivo imaging system, a product of Cambridge Research & Instrumentation) (Sragovich et al. 2019). Thus, central as well as peripheral effects are anticipated including previously observed effects on the immune system (Hacohen-Kleiman et al. 2018), impacting the microbiome (Escher et al. 2018).

### DNA extraction protocol for molecular murine fecal microbiota analysis

Fecal pellets (1–2/ mice) were collected 45 days after the beginning of treatment (NAP or DD) and were stored at − 80 °C until DNA purification procedures. The samples were thawed on ice mixed with 400 µl of sterile PBS/ sample followed by mechanical disruption of the sample using a BeadBug microtube homogenizer (Daniel Biotech, Rehovot, Israel) for 2 min at 4000 rpm. A 25-µl sample of lysozyme solution (20 mg/ml) was added, and the samples were incubated with shaking for 30 min at 37 °C on a thermo shaker racking platform. Next, 20 µl proteinase K (20 mg/ml) was included and the samples were thoroughly mixed with 400 µl of sterile lysis buffer (20 mM Tris HCl, 300 mM NaCl, 400 mM EDTA and 1% SDS) and incubated with shaking for 60 min at 56 °C on a thermo shaker racking platform. Next, 300 mg sterile zirconium beads and 150 µl of phenol was added to the samples followed by homogenization (twice for 40 s at 4000 rpm, Daniel biotech, Rehovot, Israel). Then, 150 µl of CI solution (chloroform–isoamyl alcohol 24:1) were added, mixed and submitted for centrifugation 13,000 rpm at room temperature for 5 min, Eppendorf Centrifuge 5417R (Eppendorf, Hamburg, Germany). A 600-µl volume of supernatant was removed and placed in a sterile 2 ml Eppendorf tube. 150 µl of CI solution were then included and the samples were subjected to centrifugation as above. A 400 µl of aqueous, clear top phase was removed and placed in a sterile 2 ml Eppendorf tube. Then, the 400 µl of DNA solution was mixed with 100 µl of precipitation solution (40 mM EDTA, 1.2 M sodium acetate, and 4 mg/ml glycogen). 1.3 ml of ice-cold 100% ethanol was added, and the samples were then mixed and stored over night at − 20 °C for DNA precipitation followed by centrifugation (13,000 rpm) at 4 °C. The supernatant was discarded and the pellet washed with 500 µl ice-cold 70% ethanol followed by centrifugation for 5 min at 13,000 rpm, 4 °C (this step was repeated twice). The pellet was dried in the Speed Vac (Eppendorf Concentrator 5301) for 15 min and then re-suspended in 100 µl sterile water. Next, the samples were mixed on the shaker for 10 min at room temperature. In the next step, the samples were further purified using Qiagen Purification Kit according to the manufacturer's instructions (Thermo Fisher Scientific, Hilden, Germany). DNA was eluted in 100 µl sterile water and stored at − 20 °C for long storage.

### Murine fecal microbiota analysis

In brief, DNA was further quantified using Quant-iT PicoGreen reagent (Invitrogen, UK) and adjusted to 1 ng/µl. Then, the main bacterial groups abundant in the murine intestinal microbiota including enterobacteria, enterococci, lactobacilli, bifidobacteria, *Bacteroides/Prevotella* species, *Clostridium coccoides* group, *Clostridium leptum* group, *Mouse intestinal Bacteroides* (MIB), and total eubacterial loads were determined by quantitative real-time polymerase chain reaction (qRT-PCR) with species-, genera-, or group-specific 16S rRNA gene primers (TibMolBiol, Germany) as reported previously, and numbers of 16S rRNA gene copies per nanogram DNA of each sample were assessed (Escher et al. 2018).

## Behavioral analyses

### Open field

The test provides a unique opportunity to systematically assess novel environment exploration, general locomotor activity, and anxiety-related behavior in rodents. Before executing any cognition assessing behavioral work, it is essential to recognize whether the animal behaves in a generally normal manner and to rule out abnormal physiology or motor problems such as ataxia, etc., which would affect the proper course of the behavioral experiments. The open-field apparatus is a 50  cm × 50 cm square arena, with 30 cm high walls, and all colored white. Mice were individually placed in the corner of the open field and left to explore freely for 15 min. The distance moved and time spent in the entire open field as well as in its inner defined quadrants (center, border) was recorded using the EthoVision XT video tracking system and software (Noldus Inc. Leesburg, VA).

### Social approach and social memory

A plexiglas box was divided into three adjacent chambers, each 20 cm (length) × 40.5 cm (width) × 22 cm (height), separated by two removable doors. Steel wire pencil cups [10.16 cm (diameter), 10.8 cm (height)], www.kitchen-plus.com, were used as both containment for the target mice and as inanimate objects (weights prevent the mice from overturning the cups). Experiments were conducted *on light* during the dark phase of the mouse. Target mice (males for males and females for females) were placed inside the wire cup in one of the side chambers for three 10-min sessions on the day before the test for habituation. The next day, each subject mouse was tested in an experiment with three phases, each 10 min long (measured with a simple timer): I and II, the habituation phases (ensuring no bias), and III, the experimental phase. In phase III, an empty wire cup (novel object) was placed in the center of the right or left chambers and the cup containing the target mouse was placed in the center of the other chamber. Location of the empty wire cup (novel object) and the novel mice was counterbalanced to avoid confounding side preference. The doors were then removed and a 10-min timer was initiated. The three-chamber apparatus was cleaned between mice. The social approach task was also used as habituation for the social memory task, 3 h after the first phase (3-min exposure), the mouse was placed back into the apparatus for another 3 min (second phase), during which one cup contained the familiar mouse and the other contains a novel mouse. The positions of the familiar and novel mouse during phases 1 and 2 were counterbalanced within and between groups to exclude the possibility of positional effects, but were kept the same for a given animal.

Mice were subjected to the tests as above. Mouse movement and exploratory behavior were tracked and recorded using the EthoVision XT video tracking system and software (Noldus Inc. Leesburg, VA). The discrimination capacity (social memory) was analyzed using the formula: *D*2 = (*b* − *a*)/(*b* + *a*).

### Statistical analysis

The effects of the *ADNP* deficient genotype on microbiota composition in the *Adnp*^+/–^ mouse model were statistically tested via two-way ANOVA model with interaction. Both genotype and treatment (*ADNP*, NAP) were fitted as fixed-factors, for males and females separately. The data were analyzed after applying the logarithmic transformation since measurements showed skewness to the right. Statistical significance of the main effects of treatment, genotype, and their interaction was tested via *F* test. Post hoc analysis for pairwise comparisons across treatment/genotype was performed with Fisher’s LSD for multiple comparisons. Two-way ANOVA analysis with Fisher’s LSD as post hoc was utilized for behavioral analysis. Pearson’s correlations were used for further comparisons. Further details are available in the figure and table legends.

## Results

### Sex-dependent differences in gut microbiota composition as a consequence of *Adnp* deficiency in mice can be corrected by NAP treatment

**Fig. 1:**
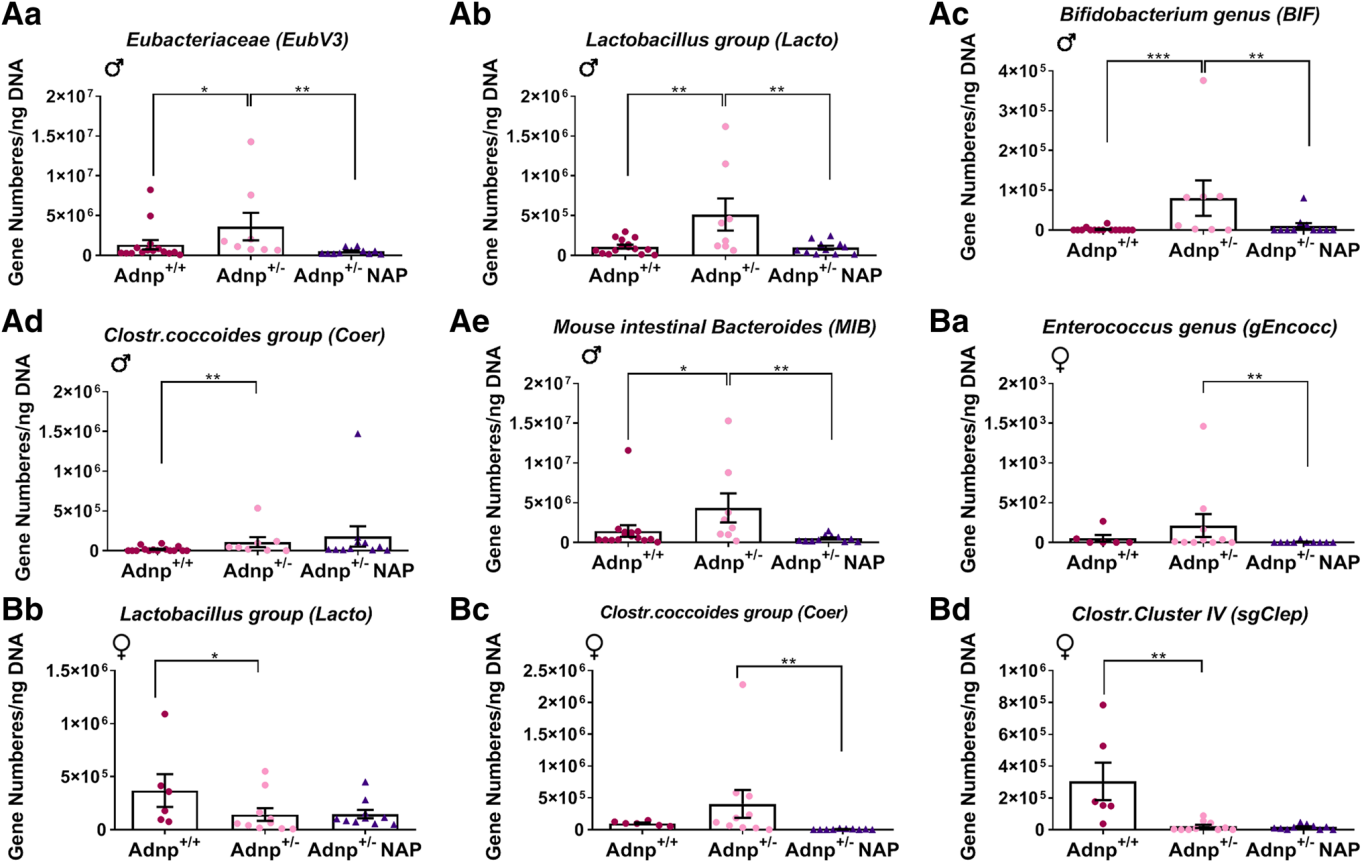
Real-time PCR analysis revealed sex-dependent differences in the bacterial genera and species in *Adnp*^+*/-*^ mice compared with *Adnp*^+*/*+^ mice, with significant amelioration following NAP treatment. Gastrointestinal flora were assessed by qRT-PCR of stool samples of 1.5-month-old male (**a**) and female mice (**b**) for nine main bacterial groups abundant in the murine intestinal microbiota (males: *Adnp*^+*/*+^ *n* = 15, *Adnp*^+*/–*^ *n* = 8, *Adnp*^+*/–*^ NAP, *n* = 11; females: *Adnp*^+*/*+^ *n* = 6, *Adnp*^+*/–*^ *n* = 10, *Adnp*^+*/–*^ NAP, *n* = 10). Results were normalized to 16S DNA. Two-way ANOVA analysis with Fisher’s LSD as post hoc revealed significant differences between DD treated *Adnp*^+*/*+^*, Adnp*^+*/–*^ and NAP-treated *Adnp*^+*/–*^ mice (*p < 0.05, **p < 0.01 and ***p < 0.001)

The following list includes all the bacterial groups tested. Labeled in bold are male *Adnp*^+/–^ genotype affected, i.e., increased as a consequence of Adnp deficiency and decreased to control levels following NAP treatment (Fig. 1A). 1] **Total eubacterial loads (EubV3)** (Fig. 1Aa), 2] Enterobacteriaceae (Entero), 3] *Enterococcus* genus (gEncocc), **4] *Lactobacillus* group (Lacto)** (Fig. 1Ab)**, 5] *Bifidobacterium*genus (BIF)** (Fig. 1Ac), 6] *Bacteroides/Prevotella* species (Bac), 7], *Clostridium coccoides* group (Coer)( Fig. 1Ad), 8] *Clostridium leptum* group (Cluster IV, sgClep), and **9] *Mouse Intestinal Bacteroides* (MIB)** (Fig. 1Ae)**.** For the *Clostridium coccoides* group, significant increases were observed in the male *Adnp*^+/−^ mice compared to male *Adnp*^+/+^ controls, whereas significant NAP-dependent decreases in *Clostridium coccoides* were observed in *Adnp*^+/−^ females. Decreased *Lactobacillus* species loads, however, were reveled in fecal samples derived from *Andp*^+/−^ females, contrasting the observed increases in respective species in male counterparts. Following NAP treatment, lower fecal *Enterococcus* genus (gEncocc) numbers were obtained in *Adnp*^+/-^ females only. Furthermore, in females, as opposed to male counterparts, *Adnp* deficiency resulted in decreased intestinal numbers of the *Clostridium leptum* group (Cluster IV, sgClep) (Fig. 1Ba–d). Bacterial groups that were neither affected by genotype nor by NAP treatment are shown in Fig. S1. Additionally, of all the bacterial groups tested, NAP treatment resulted in lower total eubacterial loads in fecal samples derived from female *Adnp*^+/+^ mice (Fig. S1), whereas higher intestinal numbers of *Clostridium coccoides* group (Coer) were observed in NAP-treated compared to placebo-treated male *Adnp*^+/+^ controls (Fig. S2).

In summary, when comparing gut microbiota composition in the currently studied *Adnp* heterozygous mice (specifically, *Adnp*^+/−^ on an ICR background), we found dramatic differences between males and females. Thus, when assessing the main bacterial group abundance in the murine intestinal microbiota including enterobacteria, enterococci, lactobacilli, bifidobacteria, *Bacteroides/Prevotella* species, *Clostridium coccoides* group, *Clostridium leptum* group, *Mouse intestinal Bacteroides* (MIB), and total eubacterial loads, essentially no overlap was observed for the *Adnp* genotype effect between males and females. While significant genotype effects were observed in five different microbiota groups in males, only two different microbiota groups were found to be significantly affected in females. Similarly, while NAP treatment reversed the male genotype deficits in four out of five genotypes affected microbiota groups, in females, it significantly corrected two genotype-dependent trending increases. Together, the data suggest a more significant effect on males and an overall NAP corrective effect (Fig. 1).

### Behavior dependent differences in gut microbiota composition

**Fig. 2:**
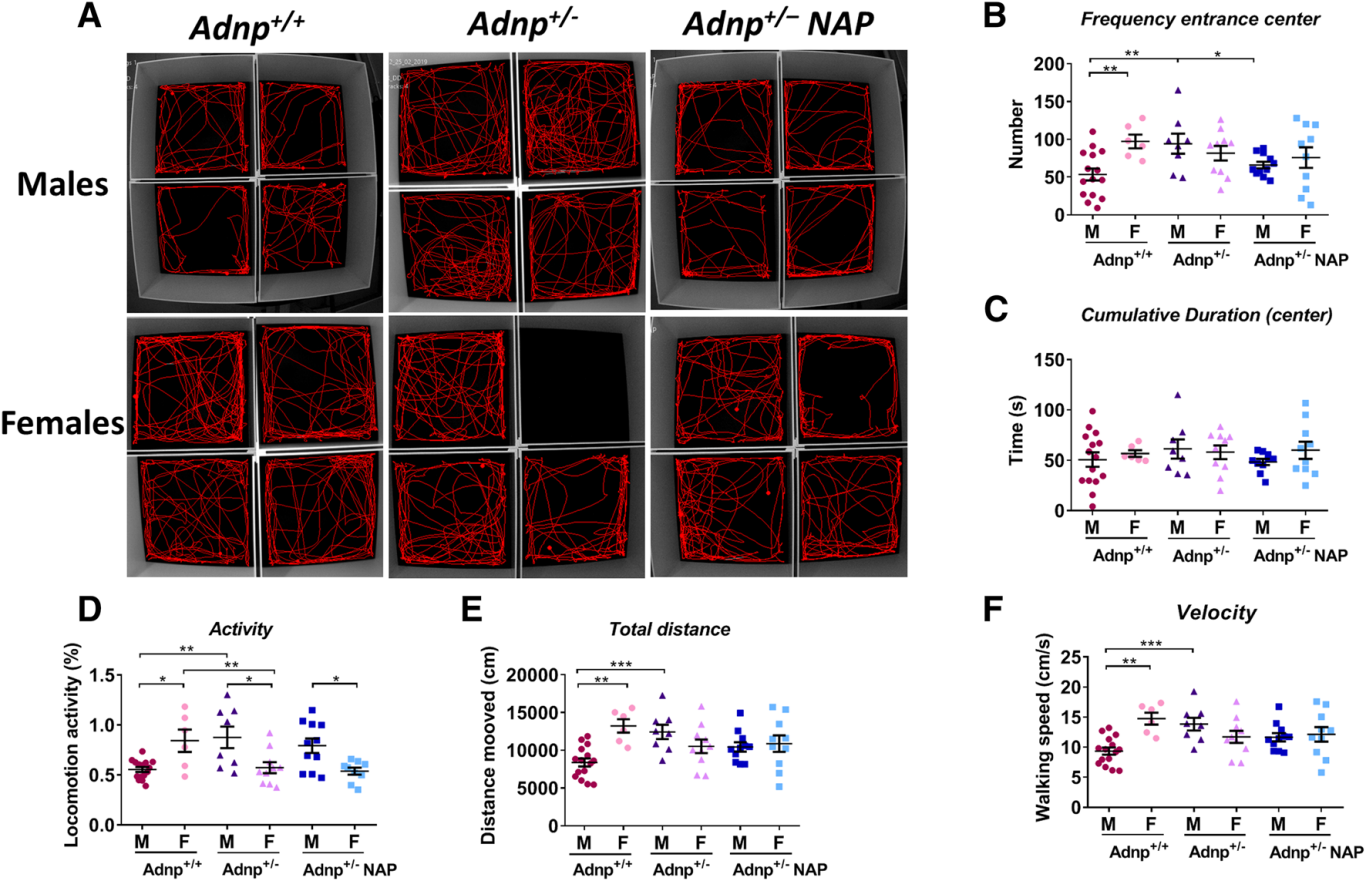
*Adnp*^+*/–*^ mice exhibited increased locomotor activity and decreased anxiety-related behaviors compared to *Adnp*^+*/*+^ mice and NAP-treated *Adnp*^+*/–*^ mice that might be at least partially attributed to increased intestinal *Bifidobacterium* and *Lactobacillus* loads. **a** Tracking visualization of the path traveled by four independent mice during the first 2 min of the open-field experiment. **b***Adnp*^+*/–*^ male mice showed significantly higher entrance frequencies to the center area of the open field compared to *Adnp*^+*/*+^ mice and NAP-treated *Adnp*^+*/–*^ mice as revealed by Two-way ANOVA analysis with Fisher’s LSD as post hoc (**p < 0.01 and *p < 0.05, respectively). Furthermore, sex-dependent differences were observed in the *Adnp*^+*/*+^ group (**p < 0.01). **c** Time spent in the center area did not reveal significant sex- or genotype-dependent differences. **d***Adnp*^+*/–*^ male and female mice were active for relatively longer periods of time (% locomotion activity time period) compared to *Adnp*^+*/*+^ mice as revealed by two-way ANOVA analysis with Fisher’s least significant difference—LSD as post hoc (**p < 0.01). Furthermore, sex-dependent differences were observed in the *Adnp*^+*/*+^ group (*p < 0.05), *Adnp*^+*/–*^ group (*p < 0.05), and NAP-treated *Adnp*^+*/–*^ mouse group (*p < 0.05). **e***Adnp*^+*/–*^ male mice traveled longer distances in the arena compared to *Adnp*^+*/*+^ mice as revealed by two-way ANOVA analysis with Fisher’s LSD as post hoc (***p < 0.001). Also, sex-dependent differences were observed in the *Adnp*^+*/*+^ group (**p < 0.01). **f***Adnp*^+*/–*^ male mice showed significant higher velocity in the arena compared to *Adnp*^+*/*+^ mice as revealed by two-way ANOVA analysis with Fisher’s LSD as post hoc (***p < 0.001). Also, sex-dependent differences were observed in *Adnp*^+*/*+^ group (**p < 0.01)

Open-field tests measuring spontaneous activity and anxiety/hyperactivity/emotional behavior revealed sex-dependent differences in the control mice (Fig. 2a–f). In particular, the frequencies of entrance to the center of the field, general activity, speed, and distance traveled were significantly higher in males as compared to female counterparts. Interestingly, these sex-dependent differences were mostly not observed in the *Adnp*^+/−^ mice, whereas general activity was even significantly decreased in female versus male the* Adnp*-deficient animals. As such, the *Adnp*^+/−^ phenotype mostly affected male behavior in the open field with increased activity in all afore-mentioned parameters. NAP treatment resulted in partial reversal of the genotype effect in males, significantly reducing the frequency of entrances to the center to the control levels. No effects, however, were observed in the cumulative duration of residence in the center of the field.

**Fig. 3:**
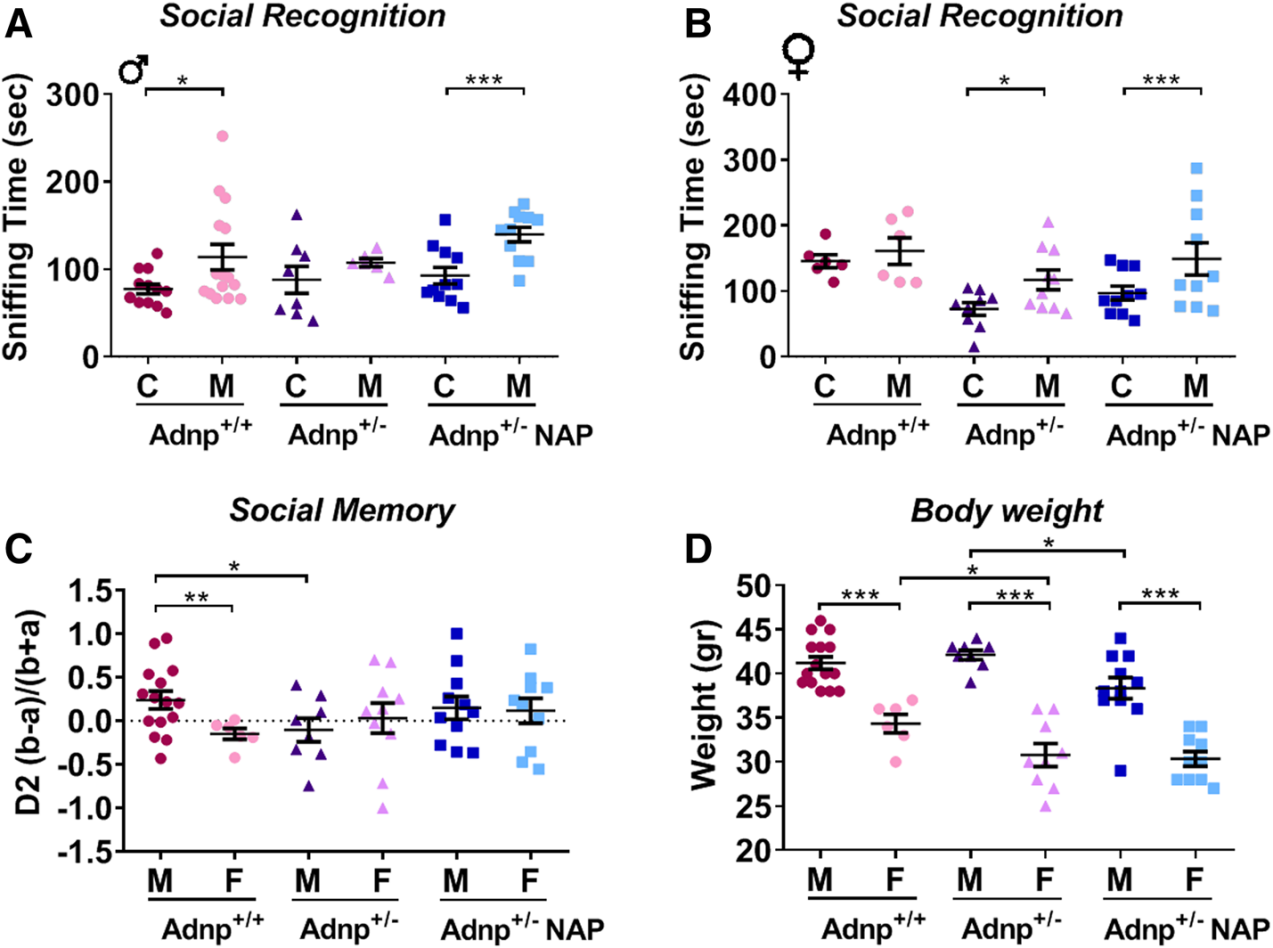
Genotype, sex, and NAP treatment-dependent effects on social recognition and body weight. Two-way ANOVA analysis with Fisher’s LSD as post hoc revealed the following differences (*p < 0.05, ***p < 0.01 and ***p < 0.001). **a**, **b** Results of the social recognition test in males and females, respectively. **c** Depicts social memory results, exhibiting sex- and genotype-dependent effect in males only**. d** Sex, genotype, and treatment-dependent effects on body weights

Extending the results to social recognition, to social memory and also to body weights (Fig. 3), revealed *Adnp*^+/−^ genotype-related autistic behavior in males (preference of cup to mouse in the social recognition test, Fig. 3a), which could be reversed upon NAP treatment. This was opposite to *Adnp*^+/+^ females showing baseline autistic behavior and contrasting social behavior in the *Adnp*^+/−^ genotype which was even more apparent after NAP treatment (Fig. 3b). For social recognition, NAP treatment was also beneficial to the *Adnp*^+/+^ mice, given autistic behavior in the *Adnp*^+/+^ female population (Fig. S3). In addition, sex-dependent differences could be observed in social memory in *Adnp*^+/+^ mice given less pronounced social memory in females as compared to male control mice. In *Adnp*^+/−^ mice, however, less distinct social memory could be assessed in males versus females (Fig. 3c), which was further decreased by NAP treatment (Fig. S3). Finally, regarding weight, apparent sex-dependent differences were observed in *Adnp*^+/+^ control mice with males being heavier than females (Fig. 3d). Whereas the *Adnp*^+/−^ female mice displayed lower body weights than female *Adnp*^+/+^ controls, NAP treatment resulted in increased weight in the male *Adnp*^+/−^ cohort.

Significant correlations were discovered among individual levels of weight, behaviors, and specific bacterial loads (Table 1). In males, most measured parameters correlated with the fecal *Bifidobacterium* genus (BIF) load, while in females, most measured parameters significantly correlated with intestinal *Clostridium* Cluster IV (sgClep) loads, with some significant behavior/bacterial loads correlations in both sexes including open-field activity [*Bifidobacterium* genus (BIF) load] and social recognition [*Clostridium* Cluster IV (sgClep)] (Table 1). Table 2 shows correlations among the different bacterial groups, with overlap between males and females and a greater number of significant correlations (20) in males versus 14 significant correlations in females.

**Table 1:**
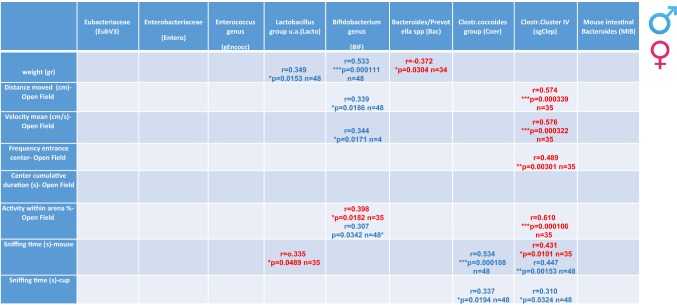
Tabular summary of changes in gut bacterial groups as well as the correlation to behavioral test results

As the data did not show normal distribution, we employed the recommended Spearman’s correlation comparing expression levels of each bacterial group relative to behavioral test results

Spearman correlation coefficients as well as the *p* values are presented in the table. Female results are presented in red and male in blue.

**Table 2:**
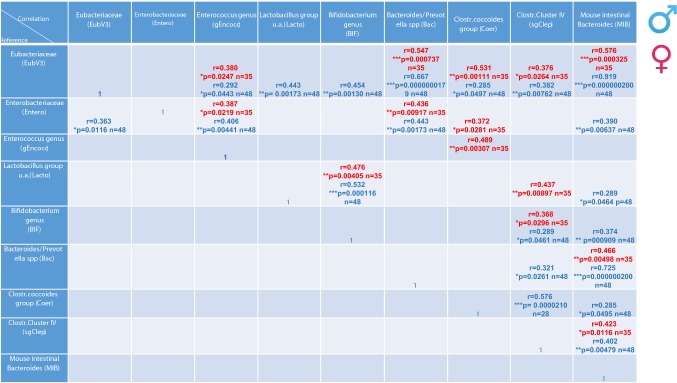
Tabular summary of dysregulated bacterial groups to each other

As the data did not show normal distribution, we employed the recommended Spearman’s correlation comparing expression levels of each bacterial group relative to other bacterial groups

Spearman correlation coefficients as well as the *p* values are presented in the table. Female results are presented in red and male in blue.

In summary, similar to the effects seen in gut microbiota composition, male behavioral parameters were found here to be more significantly affected compared to females as apparent in the open field, and in the social recognition/social memory tests and the partial correction by NAP treatment (Figs. 2 and 3). This is further demonstrated in Table 1 showing sexual dichotomy in male–female behavior in correlation with gut bacterial group differences.

## Discussion

Our present study revealed sex-dependent differences in the gut microbiota composition of *Adnp*^+/−^ mice correlating to distinct behavioral outcomes. Overall, a more robust *Adnp* genotype effect was observed in males, in the number of microbiota groups and the specificity of the effect with correction observed following the ADNP-snippet NAP treatment. Our previous studies suggested sexual dichotomy in the expression of ADNP with higher expression in the male hippocampus compared to females in mice and humans (Malishkevich et al. 2015). We then showed that ADNP interacts with the autism-linked protein eukaryotic translation initiation factor 4E (eIF4E) and further regulates it expression in males only (Malishkevich et al. 2015). In this previous study, we suggested ADNP regulation of steroid sex hormones through its chromatin remodeling functions (e.g., (Malishkevich et al. 2015; Mandel and Gozes 2007; Mandel et al. 2007)). More recent studies have shown that one of the principal regulators of circulating estrogens is the gut microbiome (Baker et al. 2017). A recent review highlights the sex differences in ASD as follows, ASD presents with social deficits and a male sex bias. ASD is also reported to result in gastrointestinal and immune changes. Rodent models of ASD result in social behavior deficits via gut-immune changes. Sex may play a role in gut-immune-brain communication (Kopec et al. 2018). Our current studies thus implicate a regulatory role for ADNP in association with gender-specific appearance of ASD.

Previously, we have shown deficient muscle activity in male *Adnp*^+/−^ mice which could be reversed following NAP treatment; furthermore, autistic behavior in the social recognition of female mice was shown to be alleviated upon NAP application, initiated at birth (Hacohen-Kleiman et al. 2018). Here, mice were treated after weaning resulting in altered behavior in the social recognition test compared to previous studies (Hacohen-Kleiman et al. 2018). Regardless, we were able to observe significant correlations of the gut microbiota composition with behavioral outcomes.

Additionally, the higher fecal *Bacteroides/Prevotella* numbers in *Adnp*^+/−^ females were shown to be associated with lower body weights (Table 1, *r* = − 0.372 *p = 0.0304, *n* = 34) which is supported by previous human data, indicating that the *Prevotella*-to-*Bacteroides* ratio correlates with body weight. Specifically, individuals with high *Prevotella*-to-*Bacteroides* ratios were more susceptible to weight loss on a diet rich in fiber (Hjorth et al. 2019).

Men and women are known to exhibit gender-specific differences in their immune system and their gut microbiota composition (Kim et al. 2020). Previously, the gut microbiota from males or females were transferred to germ-free animals of either the same or the opposing gender and, in fact, microbiota-independent gender differences in distinct immune cell repertoires were already present in germ free mice. For example, type I interferon signaling was enhanced in the intestine of germ free females (Fransen et al. 2017) compared to males. In this respect, ADNP was shown to regulate interferon expression (Medina et al. 2017). Indeed, we have originally discovered that ADNP is expressed in innate immune cell populations such as macrophages and that NAP downregulates the key inflammatory cytokines tumor necrosis factor (TNF-alpha), interleukin-16 (IL-16), and IL-12 in macrophages, suggesting that ADNP/NAP might play an important role in immune regulation, and further implying that immune regulation and neuroprotection may be mutually related processes (Quintana et al. 2006). An independent follow-up study assessed the expression of ADNP in the immune system of healthy subjects and multiple sclerosis patients (Braitch et al. 2010). Expression of the activation markers CD69 and CD154 and of IFN-gamma was also assessed. Results revealed that monocytes, B cells, and T cells, but not regulatory (CD4 + CD25 +) T cells expressed ADNP. NAP decreased the expression of CD69, CD154, and IFN-gamma in peripheral blood mononuclear cells that resulted in suppressed anti-CD3−/anti-CD28-stimulated cell proliferation. Furthermore, *ADNP* mRNA in peripheral blood mononuclear cells was reduced in multiple sclerosis patients compared to controls. Together, these data suggest that ADNP modulates the immune response, with ADNP/NAP protecting against inflammatory responses (Braitch et al. 2010). Mechanistically, major cytoplasmic targets for ADNP are the microtubule end-binding proteins, EB1 and EB3 (Oz et al. 2014). In this respect, EB1 regulates the immune synapse (Martin-Cofreces et al. 2012). Thus, T cell activation requires the growth of microtubules that is mediated by EB1. A direct interaction of the T cell receptor (TCR) complex with EB1 provides the molecular basis for EB1 activity promoting TCR encounter with signaling vesicles at the immune synapse. EB1 knockdown alters TCR dynamics at the immune synapse and prevents propagation of the TCR activation signal to the linker for activation of T cells (LAT), thus inhibiting activation of PLCgamma1 and its localization to the immune synapse (Martin-Cofreces et al. 2012). Furthermore, a directed vesicle movement between microclusters on microtubules is found in T cells, which is linked with TCR activation (Balagopalan et al. 2018). Given the ADNP/EB1 interactions, together with our current results, these findings indicate modulations of immune reactivity by ADNP concentrations. Interestingly, we found further sexual dichotomies, for example, in female mice, *Adnp*/NAP regulate splenic *Adnp* expression and further regulate genes that that impact T cell function and activity such as Mtor, and in males, NAP regulates *Mtor* (Hacohen-Kleiman et al. 2018; Myers et al. 2019). Additionally, we discovered *Adnp*/NAP regulation of *Kdm5d* (a Y chromosome gene) associated with male phenotype development (Hacohen-Kleiman et al. 2018). Antigens derived from KDM5D are thought to elicit a maternal immune response during gestation (Mendez et al. 2016), which may potentially change sexual orientation in the offspring (Bogaert and Skorska 2011). In short, ADNP regulation of chromosome Y genes like *KDM5D* may contribute in part to the sexual differences observed above.

Furthermore, in a previous study, we were able to demonstrate that NAP treatment of mice with a human gut microbiota and suffering from *Toxoplasma gondii* induced subacute ileitis resulted in dampened pro-inflammatory immune responses as compared to placebo application as indicated by lower numbers of intestinal mucosal T and B lymphocytes and lower interferon (IFN)-γ concentrations in mesenteric lymph nodes (Escher et al. 2018). Remarkably, the NAP-induced anti-inflammatory effects were not restricted to the intestinal tract, but could also be observed in extra-intestinal including systemic compartments, given that pro-inflammatory cytokines were lower in liver, kidneys, and lungs following NAP as compared to placebo application, whereas colonic and serum IL-10 concentrations were higher in the former as compared to the latter (Escher et al. 2018). The 8–9 day NAP treatment-response suggests a very fast response with an identical time-line to brain synapse (dendritic spine) protection of NAP in the *Adnp*^+/−^ mouse (Hacohen-Kleiman et al. 2018).

In general, microbiome changes were observed in ASD including the findings that children with ASD had lower percentages of *Akkermansia*, *Bacteroides*, *Bifidobacterium*, and *Parabacteroides* species, and a higher percentage of *Faecalibacterium* species in the total detected microflora compared to controls, as well as lower abundance of *Enterococcus*, *Escherichia coli*, *Bacteroides*, and *Bifidobacterium* and higher abundance of *Lactobacillus* species (Xu et al. 2019). Thus, our current results suggest that some of the microbiota groups associated with ASD are regulated by ADNP.

Other diseases of the synapse where deficiencies in ADNP or microbiome were observed include for example, Alzheimer’s disease (AD), Parkinson’s disease (PD), schizophrenia, and attention deficit hyperactivity disorder (ADHD) as follows. ADNP regulates apolipoprotein E, the major risk gene for AD, specifically in female mice (Malishkevich et al. 2015). Additionally, in humans, blood borne concentrations of ADNP correlate with intelligence (Malishkevich et al. 2016) and decrease in AD (Yang et al. 2012). Finally, somatic ADNP brain mutations in AD correlate with disease-specific tau pathology (Ivashko-Pachima et al. 2019). The most distinctive alterations in the gut microbiome composition observed in AD is the decreasing abundance of anti‐inflammatory bacterial species such as *Bifidobacterium breve* strain A1 and increasing abundance of pro‐inflammatory bacterial species such as Firmicutes and Bacteroidetes (Bostanciklioglu 2019), with both *Bifidobacterium* regulated by the *Adnp* genotype and by NAP (above and (Escher et al. 2018)) and also dysregulated in ASD (Xu et al. 2019).

Regarding PD, a potentially important link between ADNP and PD is that brain tissue from PD patients exhibits markedly reduced ADNP protein levels in neuromelanin-containing nigral neurons. Reduced ADNP levels occur early in PD (Chu et al. 2016) before reductions in catecholaminergic innervation as indicated by tyrosine hydroxylase are detected. ADNP levels are also decreased in a rat model of PD based on viral over-expression of human wild-type α-synuclein (AS), establishing a potential association between ADNP and AS-PD. Thus, down-regulation of ADNP might contribute to dopaminergic neurodegeneration via AS in PD. Complementing these findings, in vitro NAP protects against (1) dopamine (DA) and 6-OHDA toxicity in rat pheochromocytoma PC12 cells and human neuroblastoma cell lines (Offen et al. 2000), (2) AS oligomerization/aggregation (Melo et al. 2017), (3) PD mitochondria inhibited transport, (4) PD associated MT dysfunction, and (5) reduced autophagic flux (Esteves et al. 2014). NAP also protects against MT dysfunction/tauopathy in an AS-PD mouse model (Fleming et al. 2011; Magen et al. 2014). Correlating these findings with the microbiome in PD, colonization of AS-overexpressing mice with microbiota from PD-affected patients enhances physical impairments compared to microbiota transplants from healthy human donors. These findings reveal that gut bacteria regulate movement disorders in mice and suggest that alterations in the human microbiome represent a risk factor for PD (Sampson et al. 2016).

Regarding schizophrenia, we showed that ADNP is linked with the regulation of autophagy, and we further showed that this process is disrupted in schizophrenia and corrected by NAP treatment (Merenlender-Wagner et al. 2014, 2015). In this respect, an impaired gut microbiome inhibits the autophagy-mediated protein clearance process and a distinct gut microbiome composition can change the neurotransmitter levels in the brain through the vagal afferent fibers also pertinent for AD (Bostanciklioglu 2019). Current studies further evaluate gut microbiome-schizophrenia and mood disorder associations (Kanji et al. 2018).

Finally, regarding ADHD, diminished neural reward anticipation was correlated with increased *Bifidobacterium* species in the gut (Aarts et al. 2017). Given the known association between ADHD and dopamine dysregulation (Volkow et al. 2009), as well as abnormally decreased reward anticipation pathways (Scheres et al. 2007), this study speculated that dysbiosis may contribute to the clinical phenotypes of ADHD (Ming et al. 2018).

In summary, our results show, for the first time, that *ADNP* gene (haploinsufficiency (heterozygosity)) is associated with a distinct commensal gut microbiota composition in a sex-dependent manner. Mechanistically, a sexual regulation of the immune response might be invoked with direct effects on the gut microbiota composition. NAP was shown here to correct the differences in gut microbiota composition that were assessed in *Adnp*-deficient as compared to *Adnp*-intact mice. These studies pave the path to clinical evaluations of the microbiota composition in ADNP patients, coupled with cytokine/chemokine profiling as potential biomarkers for NAP (CP201) activity in clinical trials.

## Electronic supplementary material

Below is the link to the electronic supplementary material.

Fig. S1. Gut bacterial groups that did not show significant differences depending on Adnp genotype or sex (Females)

Fig. S1 Gut bacterial groups that did not show significant differences depending on Adnp genotype or sex (Males)

Fig. S2. Insignificant effects of NAP treatment on control Adnp+/+ mice

Fig. S3 Behavioral effects of NAP treatment in Adnp+/+ mice
