# Supplementary figures and images for: Microbiota changes associated with ADNP deficiencies: rapid indicators for NAP (CP201) treatment of the ADNP syndrome and beyond
Source: J Neural Transm (Vienna). 2020 Feb 18;127(2):251–63. doi: 10.1007/s00702-020-02155-5 (PMC7035218; doi:10.1007/s00702-020-02155-5)

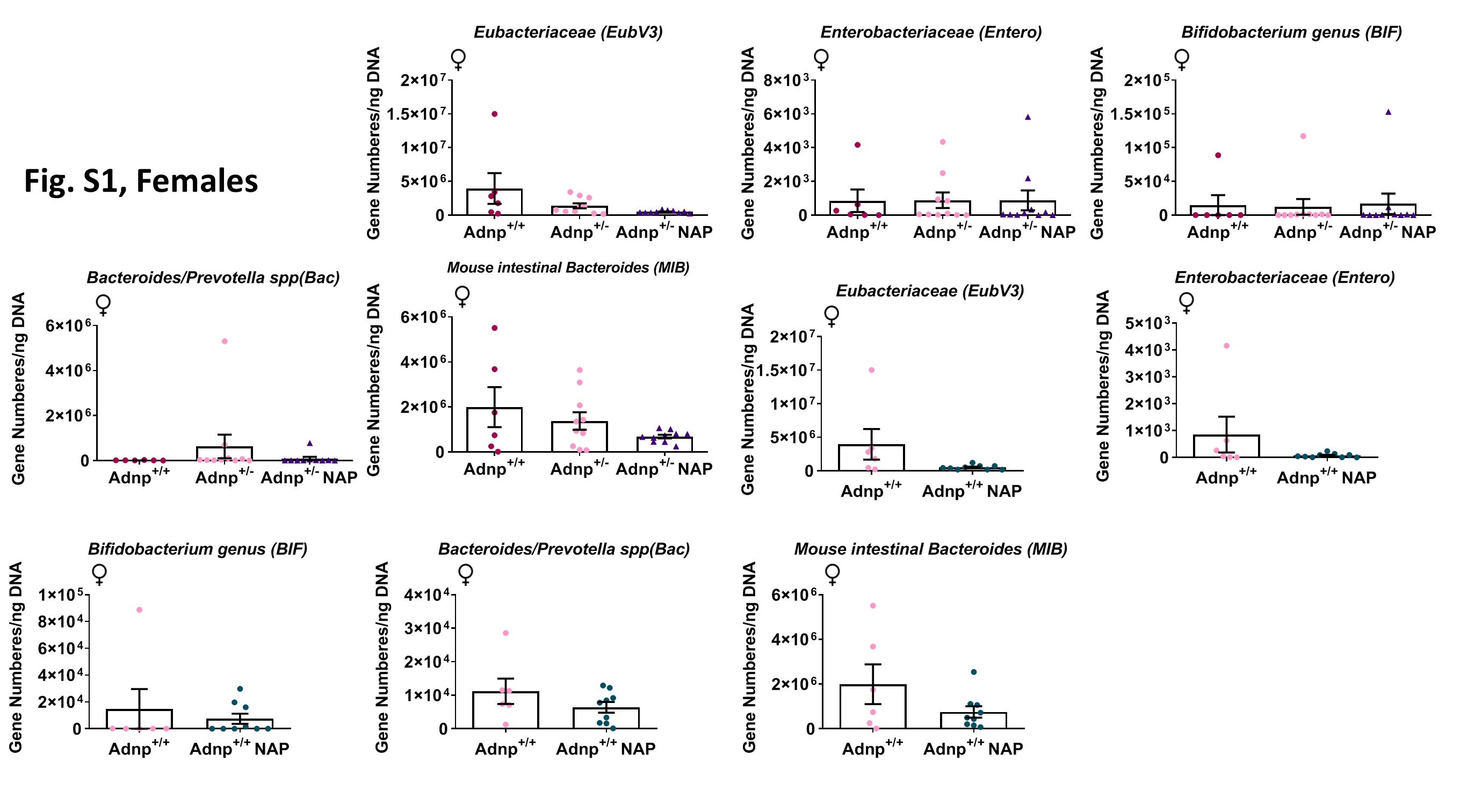

Supplement: Supplementary file 1 — Fig. S1. Gut bacterial groups that did not show significant differences depending on Adnp genotype or sex (Females) [file 702_2020_2155_MOESM1_ESM.tif]

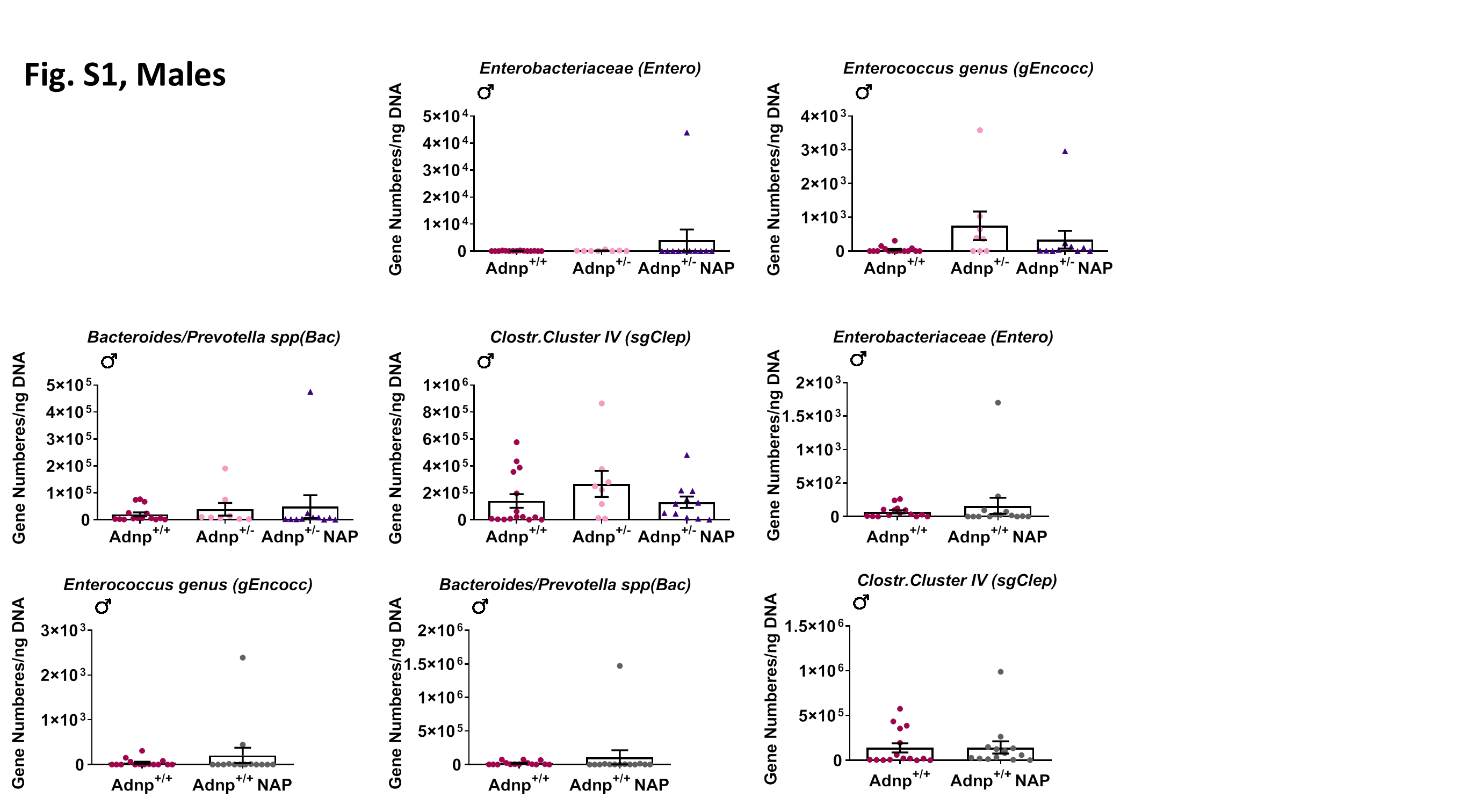

Supplement: Supplementary file 2 — Fig. S1 Gut bacterial groups that did not show significant differences depending on Adnp genotype or sex (Males) [file 702_2020_2155_MOESM2_ESM.tif]

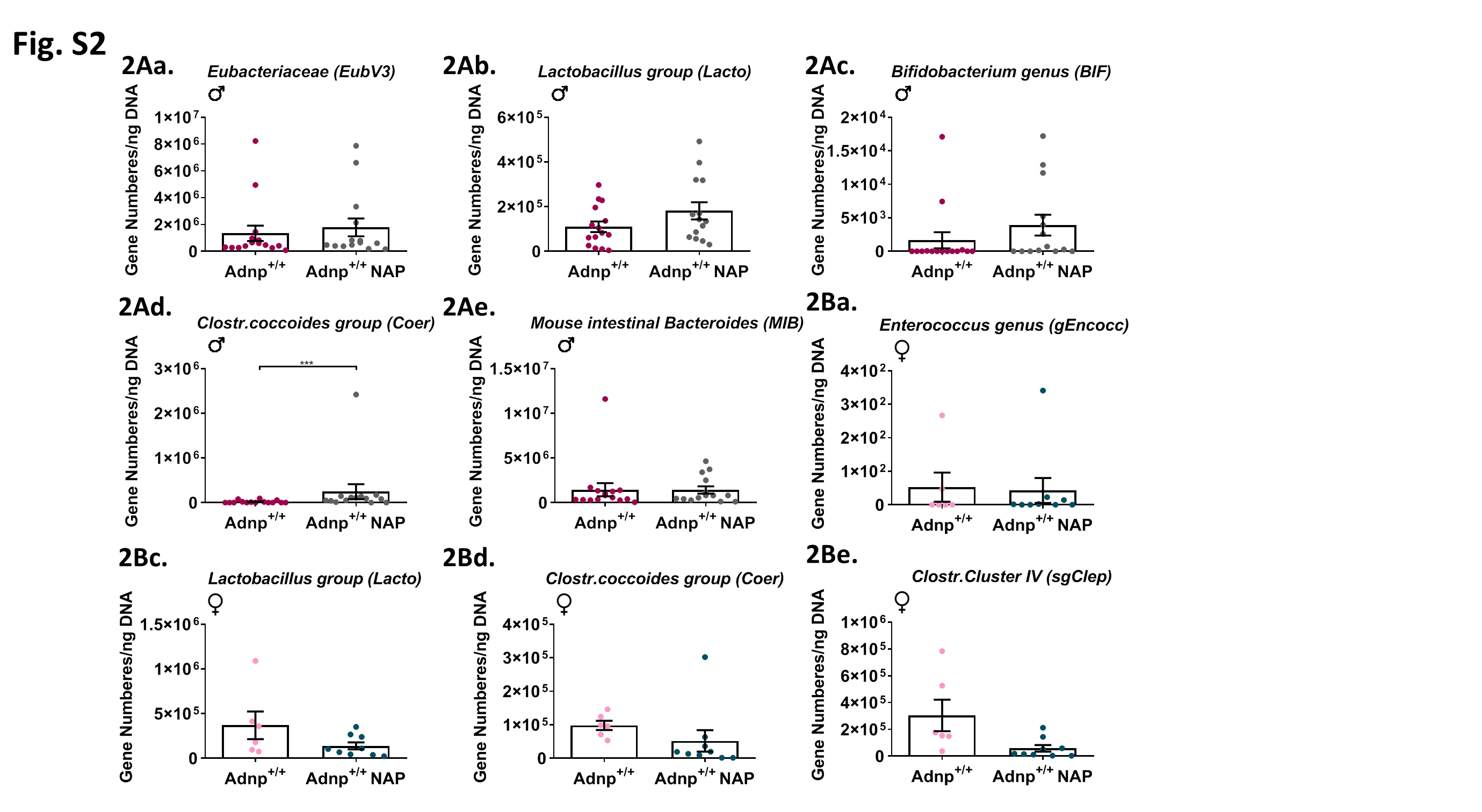

Supplement: Supplementary file 3 — Fig. S2. Insignificant effects of NAP treatment on control Adnp+/+ mice [file 702_2020_2155_MOESM3_ESM.tif]

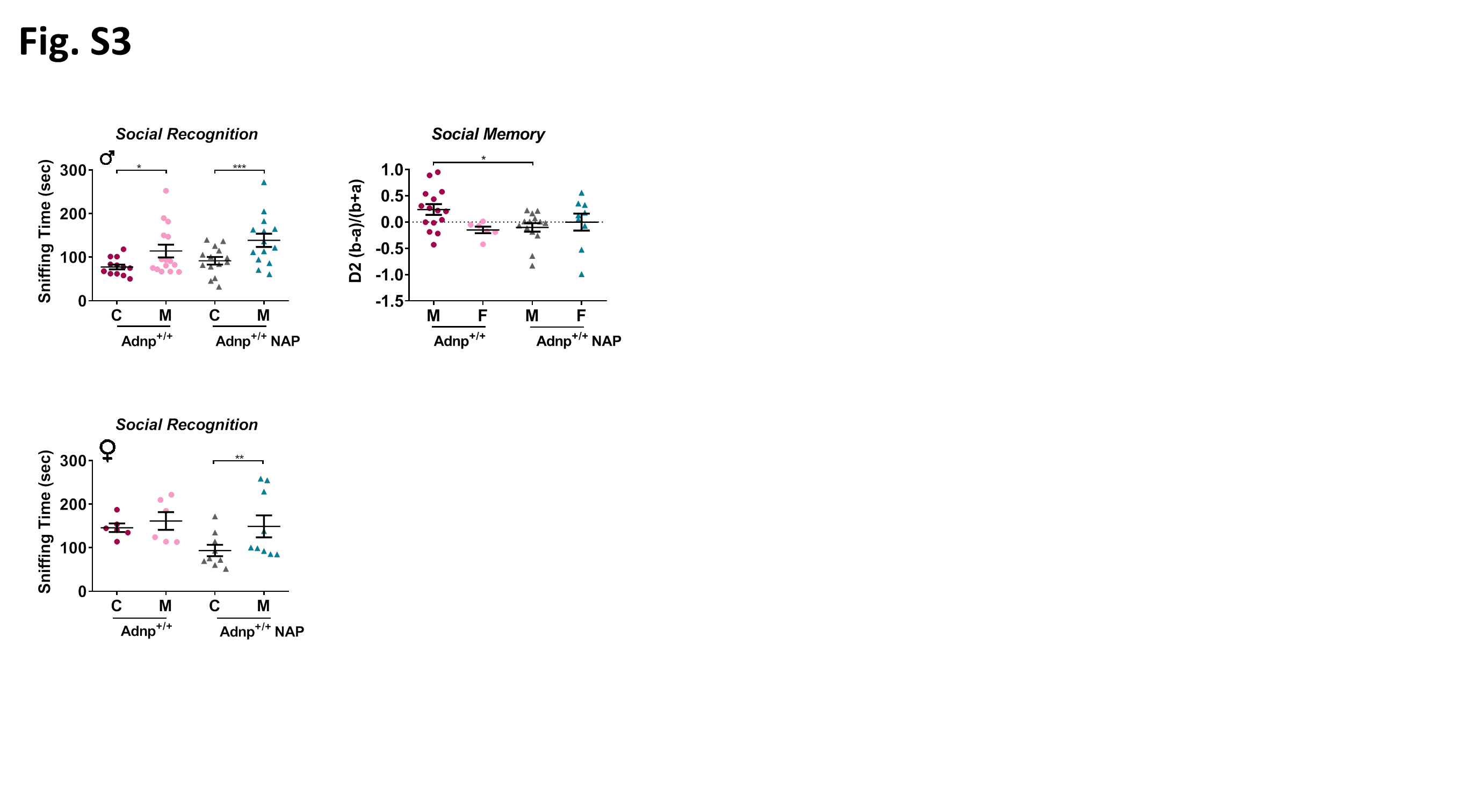

Supplement: Supplementary file 4 — Fig. S3 Behavioral effects of NAP treatment in Adnp+/+ mice [file 702_2020_2155_MOESM4_ESM.tif]
